# From the Free Ligand to the Transition Metal Complex: FeEDTA^–^ Formation Seen at Ligand K-Edges

**DOI:** 10.1021/acs.inorgchem.2c00789

**Published:** 2022-06-28

**Authors:** Sebastian Eckert, Eric J. Mascarenhas, Rolf Mitzner, Raphael M. Jay, Annette Pietzsch, Mattis Fondell, Vinícius Vaz da Cruz, Alexander Föhlisch

**Affiliations:** † Institute for Methods and Instrumentation for Synchrotron Radiation Research, Helmholtz-Zentrum Berlin für Materialien und Energie GmbH, 12489 Berlin, Germany; ‡ Institut für Physik und Astronomie, Universität Potsdam, 14476 Potsdam, Germany

## Abstract

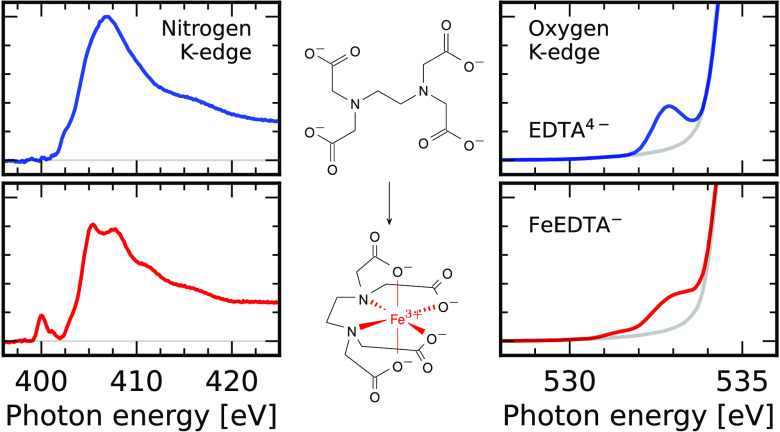

Chelating agents are an integral part of transition metal complex chemistry with broad biological and industrial relevance. The hexadentate chelating agent ethylenediaminetetraacetic acid (EDTA) has the capability to bind to metal ions at its two nitrogen and four of its carboxylate oxygen sites. We use resonant inelastic X-ray scattering at the 1s absorption edge of the aforementioned elements in EDTA and the iron(III)-EDTA complex to investigate the impact of the metal–ligand bond formation on the electronic structure of EDTA. Frontier orbital distortions, occupation changes, and energy shifts through metal–ligand bond formation are probed through distinct spectroscopic signatures.

## Abstract 2

Metal-ligand interactions between the hexadentate chelating agent EDTA and iron(III) ions in the high spin iron(III)-EDTA complex are probed using ligand K-edge X-ray absorption and resonant inelastic X-ray scattering. Distinct spectral signatures of metal–ligand bond formation e.g. frontier orbital distortions, occupation changes, and energy shifts are detected at the coordinating sites. The absence of strong spin orbit coupling effects allows for direct interpretation based on TD-DFT based spectrum simulations.

## Introduction

Among chelating agents the deprotonated ethylenediaminetetraacetic anion (EDTA^4–^) holds a prominent role due to its broad application spectrum. It comprises medical applications, for example, treatment of the autoimmune disease psoriasis,^1^ as well as acute heavy metal poisoning,^2^ dental root canal cleaning^3,4^ and the use as an anticoagulating additive^5^ with recent attention in the context of SARS-CoV-2 antibody detection.^6^ Furthermore, EDTA^4–^ is widely used in cosmetic products as well as in the context of industrial bleaching processes. The large quantities consumed in these industrial branches also pose risks due to potential environmental effects like metal ion mobilization from sediments, eutrophication, enhancement of heavy metal uptake in plants and impact on photosynthetic activity, metal ion deficiency in cells, as well as adverse developmental and reproductive effects in mammals.^7^ Recently, incorporation of EDTA in organic frameworks has been discussed in the context of wastewater treatment.^8^

**Figure 1:**
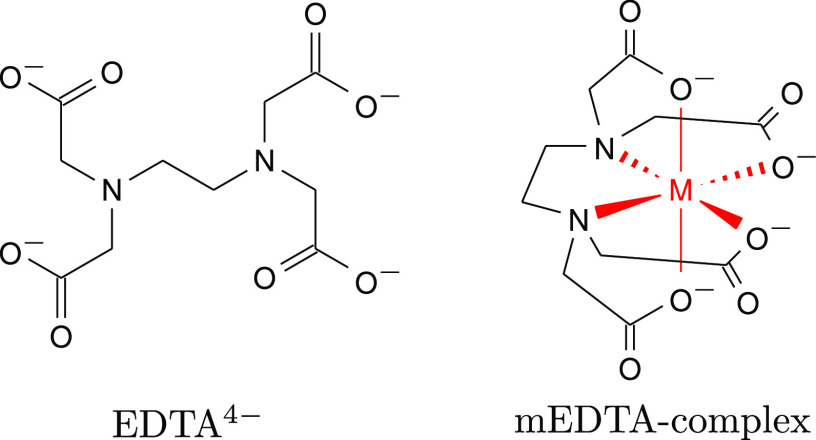
Coordination of the nitrogen and oxygen sites of EDTA^4–^ with metal ions forming mEDTA-complexes.

The hexadentate EDTA^4–^ is capable of binding a multitude of metal(III) ions *m*, through coordination with its nitrogen and oxygen sites as illustrated in Figure 1. Especially among the 3d-transition metals, the *m*EDTA complexes exhibit large variations in electronic structure altering orbital populations and spin states. In a recent study, Yuan et al. accessed the full valence electronic structure through binding energies from valence band photoemission spectra and investigated the aforementioned large variation for different metal centers (*m* = Al, Sc, V–Co).^9^ Here, we illustrate how soft X-ray absorption and resonant inelastic X-ray scattering at the ligand K-edges can be used to further dissect the electronic structure through their orbital and spin sensitivity and thereby reveal covalent bonding mechanisms upon *m*EDTA complexation. We study exemplarily the impact of the metal ligand bond formation with element specificity at all coordinating sites of EDTA^4–^ upon binding iron(III) ions, forming FeEDTA^–^. Its nitrogen as well as oxygen atoms of its four carboxylate-groups coordinate with metal centers via their lone-pair orbitals. Their strong local 2p character makes K-edge RIXS the ideal probe for the bonding channels upon complex formation. This scheme has proven to yield detailed information on the electronic structure of closed-shell complexes.^10,11^ Here, we utilize the considerable gain in information content for investigations of high spin complexes where the electronic structure information in L-edge spectra is obscured by the spin–orbit coupling induced multiplet structure of the absorption profile. In our case, the resulting necessity for an orbital sensitive probe becomes even more evident considering the striking resemblance between the L-edge X-ray absorption spectrum of FeEDTA^–^ (see SI) and the one of free iron-(III) ions in alcohol solution^12^ which impedes direct interpretation due to the absence of distinct spectral signatures of the metal–ligand bond.

## Materials and Methods

### Experiment

The X-ray absorption and RIXS measurements were performed in the nmTransmission NEXAFS endstation at the beamline UE52_SGM^13^ and the EDAX experiment at the beamline UE49_SGM^14^ of the synchrotron BESSY II. EDTA disodium salt dihydrate and EDTA ferric sodium salt (NaFeEDTA) were dissolved in deionized water at a concentration of 0.1 M for the X-ray absorption measurements. The pH of the EDTA solution was adjusted to 10 to fully deprotonate the carboxyl groups and generate EDTA^4–^ using sodium hydroxide. The RIXS measurements were performed at higher concentrations of 0.5 M for EDTA^4–^ and 0.2 M for FeEDTA^–^. The solutions were injected into the experimental vacuum chamber in a liquid flat-jet system allowing for transmission X-ray absorption measurements. A cylindrical jet was used for the RIXS measurements. The details of the experimental end-stations are described in detail by Fondell et al.^15^ and Kunnus et al.^16^ The X-ray absorption measurements were performed with linear horizontally polarized X-ray radiation, whereas vertical polarization was used in the RIXS measurements if not denoted differently. A non-negligible background of solvent emission was subtracted from the oxygen K-edge RIXS spectra.

### Theory

The geometry of EDTA^4–^ and FeEDTA^–^ were optimized at the DFT level of theory using the PBE0 exchange correlation functional. Interaction with the surrounding water environment was treated implicitly using a conductor like polarizable continuum model.^17^ The def2-TZVP(-f)^18^ and the def2/J^19^ auxiliary basis sets were used. The atom-pairwise dispersion correction with the Becke-Johnson damping scheme (D3BJ) was utilized.^20,21^ Presented orbital isosurfaces are shown for an isovalue of 0.1 and 0.05 for EDTA^4–^ and FeEDTA^–^, respectively. A coordinating water molecule was considered explicitly for the simulations of FeEDTA^–^. The X-ray absorption spectra were computed using the core–valence separation scheme, including only the nitrogen or oxygen 1s orbitals in the donor- and all virtual orbitals in the TD-DFT acceptor space. The RIXS spectrum simulations were performed in the RSA-TD-DFT framework described by Vaz da Cruz et al.,^22^ allowing for excitations from the nitrogen and oxygen 1s, as well as all occupied valence orbitals into the 20 energetically lowest virtual orbitals. For the closed shell EDTA^4–^ and the open-shell sextet FeEDTA^–^ simulations, the TD-DFT schemes were applied to the orbitals from a restricted and an unrestricted reference, respectively. RIXS spectra were mostly simulated in the localized approximation, where only individual 1s core orbitals are part of the TD-DFT donor space and the spectra were computed as the sum of spectra for the individual core holes localized at the oxygen and nitrogen sites. Thereby interference effects are neglected, which is the an acceptable approximation for resonant excitations. Spectra at strongly detuned excitation conditions are more strongly affected by interference effects. Therefore, the EDTA^4–^ oxygen edge detuning series of spectra are also compared to simulations in the delocalized approximation including all oxygen 1s core-orbitals. The absorption spectra were shifted by 11.6 eV at the nitrogen and 13.2 eV at the oxygen edge for optimal alignment with the experimental data. Geometry optimizations and TD-DFT simulations were performed using the ORCA quantum chemistry package of version 5.0.1.^23^ The RIXS spectrum simulations were performed based on the Kramers-Heisenberg formula using transition dipoles extracted from the Orca output with the Multiwfn software.^24^ The scheme is based on a pseudo wave function ansatz, which is described in more detail by Nascimento et al.^25^

## Results and Discussion

**Figure 2:**
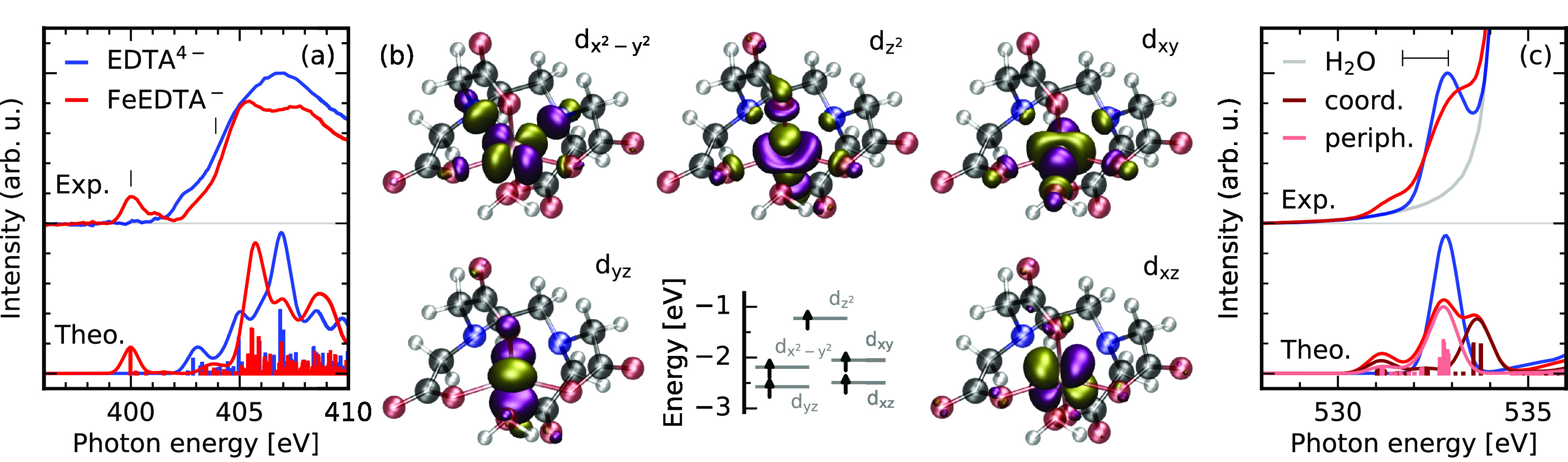
Accessing the metal–ligand bond at ligand 1s absorption edges. The singly occupied d_*x*^2^–*y*^2^_, d_*xy*_, and d_*z*^2^_ derived frontier orbitals (b) with Fe–N and Fe–O σ*-character are accessible through absorption lines in the nitrogen (a) and oxygen (c) K-edge absorption spectra of FeEDTA^–^. The d_*yz*_ and d_*xz*_ orbitals (b) can be additionally probed at the oxygen K-edge (c) through their overlap with the carboxylate π-system. Contributions of the coordinating (dark red) and the peripheral (light red) oxygen sites are shown separately. The legend in (a) holds also in (c). Excitation energies for the RIXS measurements presented in Figures 3 and 4 are also indicated.

We start by analyzing the nitrogen K-edge X-ray absorption spectrum of EDTA^4–^ presented in Figure 2a. It exhibits a broad σ* absorption band for photon energies exceeding 401 eV, which are characteristic to nitrogen K-edge absorption profiles of systems with local tetrahedral symmetry imposed by the sp^3^-hybridization. We also detect a distinct shoulder in the pre-edge region between 401 and 403 eV. Such pre-edge transitions are common in nitrogen K-edge absorption spectra of triply coordinated nitrogen sites^26−29^ for which the effective *C*_3*v*_ selection rules in the tetrahedral arrangement are softened, yielding sizable intensity for pre-edge transitions into the low lying unoccupied orbitals close to the Fermi level.

The onset of the main σ*-absorption band of FeEDTA^–^ is blue-shifted with respect to EDTA^4–^ from 401 to 402 eV. The shift can be rationalized considering the σ-donation at the Fe–N bond, which abstracts lone-pair electron density from the nitrogen site which stabilizes the 1s core level due to the reduced screening of the core-potential. This effect is analogous to the well-established protonation-dependent core level shift of nitrogen 1s absorption spectra.^29−34^ Furthermore, the spectrum of FeEDTA^–^ exhibits two additional pronounced absorption resonances at photon energies between 399 and 402 eV. The resonance at 400 eV exhibits the highest intensity whereas an energetically higher lying resonance is seen as a shoulder at 401 eV. To understand the origin of these resonances as well as their intensity, we consider the orbitals depicted in Figure 2b which are attributed to the simulated transitions in this energy range, as well as the orbital diagram describing the ligand field splitting in pentagonal bipyramidal symmetry in Figure 2b.

Even though EDTA^4–^ is an hexadentate chelating agent, its short ethylene backbone does not permit bond angles of 90° as commonly seen for smaller or very flexible ligands resulting in nearly octahedral complexes. Instead, the bonding angles in the *xy*-plane are on the order of 70° letting a water molecule coordinate with the metal center in aqueous solution environments.^9^ Hence, the coordinating water molecule is included in all simulations. Because of the only moderate ligand strength, the FeEDTA^–^ complex has a sextet ground state. It has been shown for octahedral *d*^5^ configurations, that the *t*_2*g*_ vacancy has sizable overlap with the nitrogen 1s core orbital through the covalent metal–ligand interaction.^35−37^ Likewise, the two detected nitrogen 1s absorption resonances for FeEDTA^–^ below the σ*-band can be assigned to orbitals with d_*xy*_ and d_*x*^2^–*y*^2^_ character illustrated in Figure 2b. Especially the energetically lower lying d_*x*^2^–*y*^2^_ orbital is oriented along the Fe–N bond and thus mixes strongly with the nitrogen sp^3^-hybridized orbital forming a σ*-orbital with a high degree of nitrogen 2p character. Therefore, the intensity of the transition at 400 eV exceeds the one at 401 eV which originates from absorption into the orbital with dominant d_*z*^2^_ character, but weaker nitrogen 2p contribution.

Because of their nodes in the *xy*-plane as well as their nodes on the Fe–N bond axes, the d_*xz*_ and the d_*yz*_ orbitals do not exhibit any mixing with the group of nitrogen 2p orbitals. Therefore, transitions into these frontier orbitals are not accessible at the nitrogen K-edge. However, their mixing with the carboxylate π*-systems makes them accessible at the oxygen K-edge. We thus focus on the absorption spectrum in the energy region up to 534 eV just below the solvent absorption onset presented in Figure 2c.

The spectrum of EDTA^4–^ exhibits a single absorption line on top of the solvent absorption background. The simulations let us assign this absorption line to transitions between the nearly degenerate carboxylate oxygen 1s orbitals and the carboxylate π*-system. Similarly to the nitrogen K-edge spectrum, FeEDTA^–^ complex formation opens additional absorption resonances at lower photon energies. In contrast to the measurements at the nitrogen K-edge, the energetically lowest transitions also involve excitation into the d_*xz*_ and d_*yz*_ orbitals which have partial oxygen 2p character. The d_*yz*_ orbital exclusively mixes with the oxygen lone-pair orbitals in the plane of the carboxylate groups binding the iron center along the *z*-axis and the out-of plane lone-pair of the water molecule. In contrast, the d_*xz*_ orbital mixes with the π-system of all carboxylate groups of the EDTA^4–^-ligand and has no amplitude at the oxygen site of the coordinating water molecule.

Energetically higher lying transitions closer to the π*-resonance of EDTA^4–^ correspond to dominant excitations into the orbitals with d_*xy*_, d_*x*^2^–*y*^2^_ and d_*z*^2^_ character, which are also accessible at the nitrogen edge. These orbitals in the *xy*-plane also have Fe–O σ*-character as can be seen in Figure 2b. For energies beyond the EDTA^4–^ π*-resonance mainly excitations into EDTA based orbitals with small iron 3d admixture increase the absorption cross-section. We dissect the absorption profile of FeEDTA^–^ at the oxygen K-edge in Figure 2c into contributions from sites coordinating with the iron center (dark red) and the peripheral sites (light red). This analysis shows that the ligand field orbitals are accessible from both sets of oxygens but that the energy of the strongest absorption line remains unaltered for the peripheral sites whereas a strong blue shift is present for the coordinating sites. In this context, one has to note that the absorption resonance of the oxygen site of a coordinating water molecule in the considered energy range has comparably weak intensity and largely overlaps with the π* absorption band of the peripheral oxygens making this site spectroscopically rather inaccessible.

**Figure 3:**
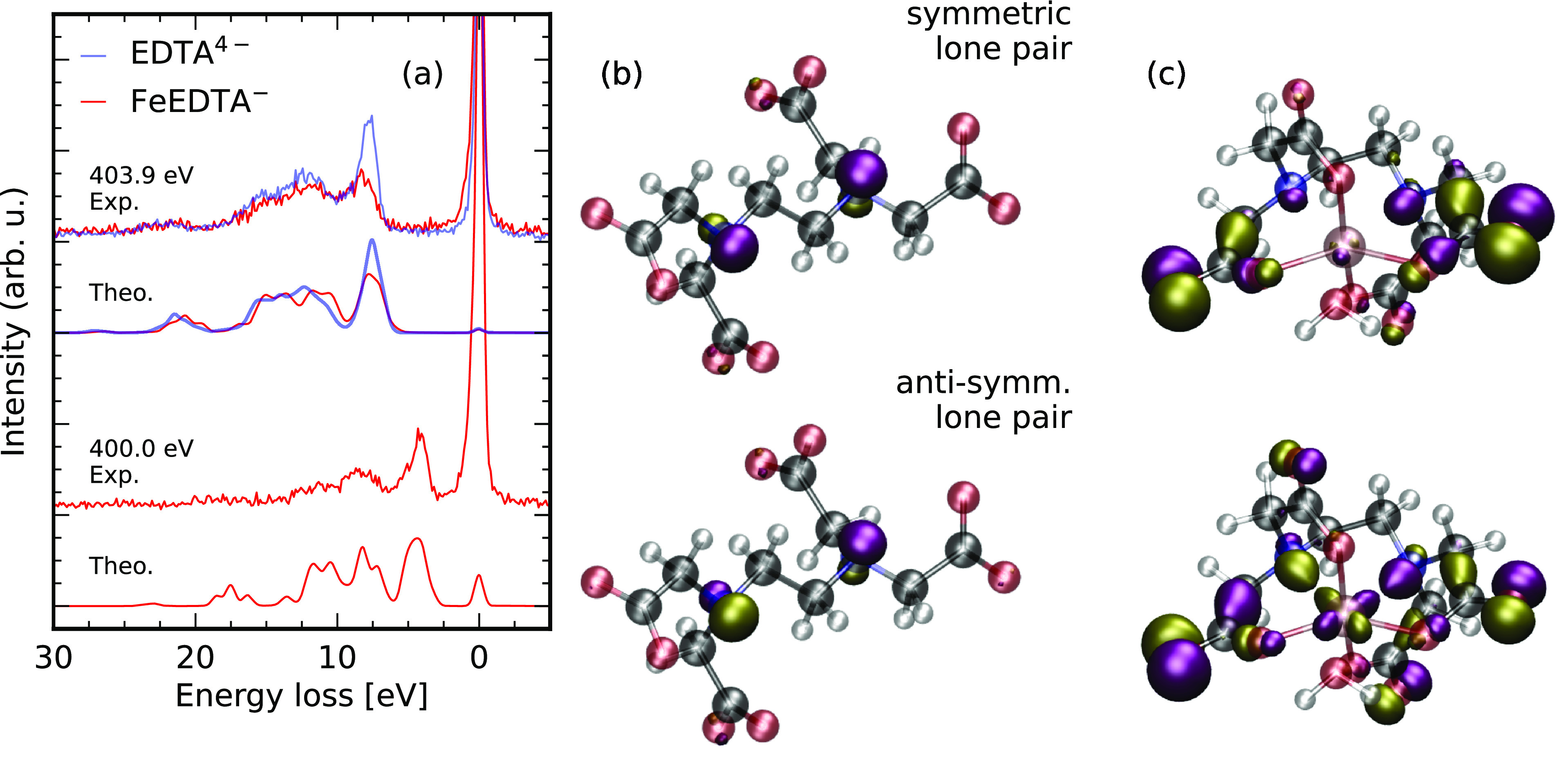
Probing the Fe–N bond in FeEDTA^–^ through nitrogen K-edge RIXS. The RIXS spectra through the ligand field orbital related resonance at 400 eV and the σ* resonance of the EDTA backbone at 403.9 eV (a) yield access to the covalent interaction at the iron and the nitrogen site through the emission from nitrogen lone-pair orbitals of EDTA^4–^ (b) of which the symmetric linear combination mixes with the metal d_*xy*_-orbital whereas the antisymmetric one forms the chemical bond with the metal d_*x*^2^–*y*^2^_-orbital (c).

Having discussed the formation of partially occupied or unoccupied molecular orbitals upon EDTA-complexation based on their K-edge absorption signatures, we will now focus on the manifold of occupied orbitals and analyze changes therein using resonant inelastic X-ray scattering through the previously discussed resonances. We consider the nitrogen K-edge RIXS spectrum of EDTA upon excitation at the rising flank of the σ*-band at 403.9 eV in Figure 3a. It exhibits a distinct emission line at an energy loss of 7.5 eV, which corresponds to emission from the occupied, nonbonding nitrogen sp^3^-lone-pair orbitals depicted in Figure 3b. One should note the existence of nearly degenerate occupied orbitals with equal and inverse parity at the two nitrogen sites as the two highest occupied molecular orbitals. The slight asymmetry of the emission line toward higher losses is also related to an orbital with local nitrogen lone pair character but larger amplitude in the planes of the carboxylate groups, reducing the corresponding spectral intensity related to decay from this orbital in the RIXS spectrum. The broad emission band between 9 and 18 eV energy loss corresponds to emission from N–C σ-orbitals changing sign at the nitrogen sites. They thus have local nitrogen 2p character which yields sizable emission intensity. Their delocalization across the EDTA σ-backbone results in their reduced intensity compared to the lone-pair emission line. The emission band at energy losses beyond 18 eV is related to a set of deep lying σ-bonding orbitals which span multiple bonds with a reduced number of nodes. In the framework of canonical orbitals in tetrahedral bonding environments, these orbitals are derived from atomic orbitals with s-character, whereas the band between 8 and 18 eV loss reflects emission from mostly p-derived orbitals. Nodes at the nitrogen atoms for both sets are essential to yield emission intensity in the K-edge RIXS spectra.

Having assigned the nitrogen K-edge RIXS spectroscopic fingerprints in EDTA^4–^, we can study the impact of the metal–ligand bond formation, by comparison of the RIXS spectra of EDTA^4–^ and FeEDTA^–^ on the rising flank of the σ* resonance at 403.9 eV in Figure 3a. The spectra are normalized to the scattering intensity for energy losses larger than 10 eV. A justification for the normalization will be given later. We detect a severe relative reduction of scattering intensity of the lone-pair emission with respect to the deeper lying σ-band in FeEDTA^–^ compared to EDTA^4–^ as a result of the σ-donating interaction. Both the experimental data and the simulations show that the lone-pair line does not only reduce intensity, but exhibits a broadening due to the different covalent interaction of nitrogen lone-pair orbitals with the iron 3d-orbitals. Emission channels in both spin-manifolds contribute to the discussed spectrum, as excitations of both spin-up and down 1s electrons into the totally unoccupied σ* orbitals are possible. This complicates direct orbital assignment based on the simulated transitions, as the spatial part of both spin subsets of orbitals in the probed transitions vary due to the used unrestricted Kohn–Sham framework.

**Figure 4:**
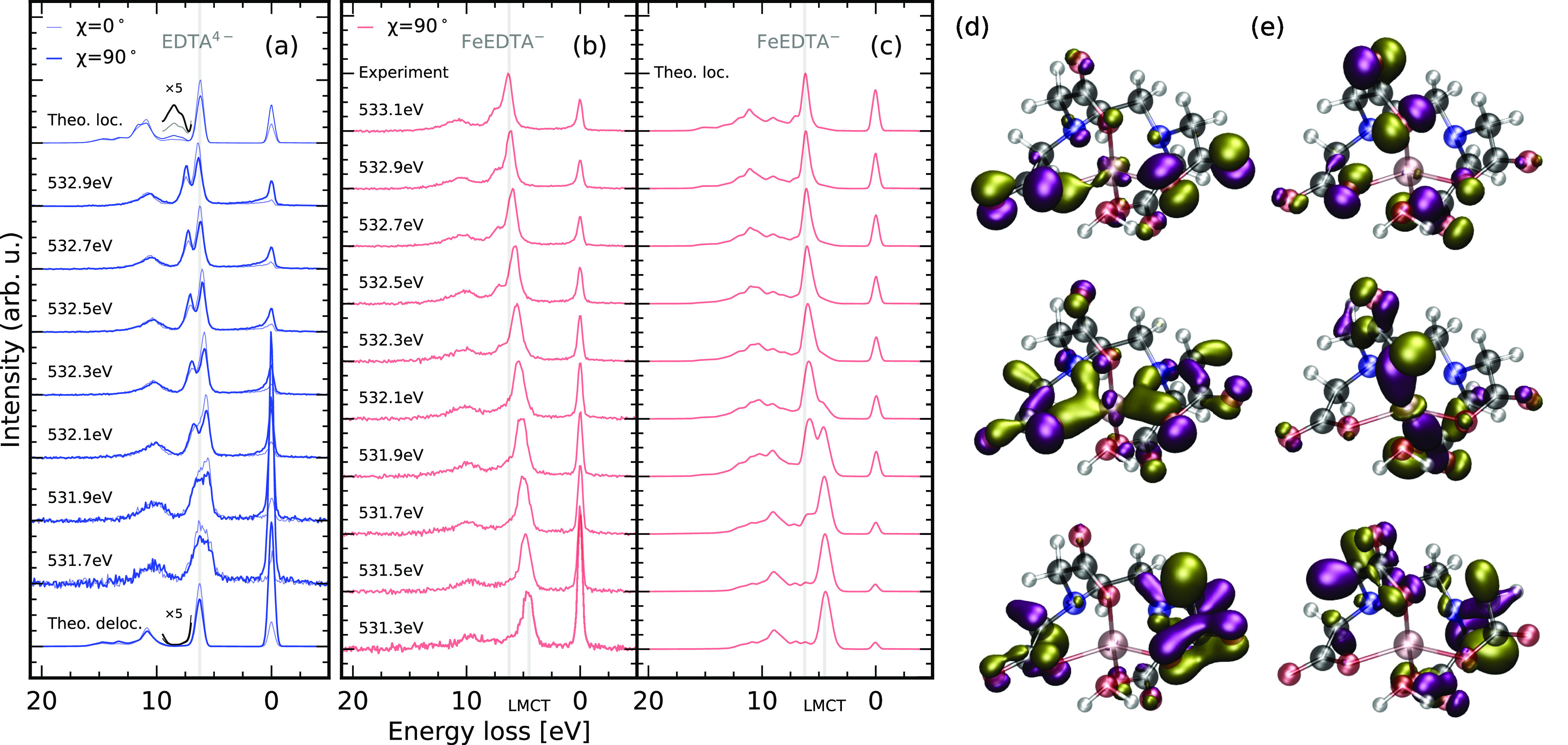
Bonding mechanisms involving the oxygen sites in FeEDTA^–^ through oxygen K-edge RIXS. RIXS spectra through the through oxygen 1s → π*-transtions. Excitation energy and polarization dependent RIXS spectra of EDTA^4–^ (a) and FeEDTA^–^ (b, experimental) and (c, theoretical, localized approximation) allow to investigate covalent σ- and π-bonding schemes at the FeEDTA^–^ oxygen sites by orbital assignment for equatorial (d) and axial (e) bonds.

To simplify the assignment and thereby allow a closer analysis of the spectral effects, we consider scattering through the absorption resonance opened upon complex formation at 400 eV. Here, the 1s core-hole created in the absorption step is within the spin-down set of orbitals, as all metal d levels are singly occupied. This is induced by the moderate strength of the EDTA^4–^ ligand. Because of the dipole selections rules of RIXS and the absence of strong spin orbit coupling in the intermediate state for K-edge RIXS, only decay channels toward states characterized by excitations between spin-down orbitals contribute spectral intensity. The corresponding RIXS spectrum exhibits the lone pair emission band shifted toward an energy loss of 4.2 eV reflecting the presence of the ligand field orbitals within the HOMO–LUMO gap of EDTA^4–^. The band is broader and more asymmetric than it is in the previously considered spectrum of EDTA^4–^. Analyzing the contribution of different decay channels to the spectrum at the resonance, it becomes obvious that the flank of the emission band toward lower energy loss is related to final states characterized by excitations from an orbital with nitrogen lone-pair character which exhibits strong covalent admixture from the iron d_*x*^2^–*y*^2^_ orbital. As the d_*x*^2^–*y*^2^_ changes signs along the N–Fe bond, only orbitals with inverse local parity at the nitrogen atoms exhibit sizable covalent interaction with this 3d orbital. Transitions related to the symmetric linear combination of lone-pair orbitals which exhibit minor d_*xy*_ admixture are slightly shifted toward higher energy losses in the emission band. The deeper lying transitions are related to the σ-orbitals across EDTA^4–^ which exhibit no significant mixture with the iron 3d orbitals. This absence of any sizable metal character upon complexation in the deep lying σ-bands with p- and s-derived atomic contributions justifies the normalization of the spectra to the intensity of the related emission bands. The threshold of 10 eV for the energy interval used for the normalization was chosen to exclude the tail of the lone pair emission line from the interval.

Scattering through the oxygen π* resonances allows us to investigate the involvement of specific occupied orbitals in bonding at the oxygen sites of the FeEDTA^–^ complex. The excitation energy dependent emission spectra of both EDTA^4–^ and FeEDTA^–^ are presented in Figure 4. To rationalize the origin of individual emission lines in the RIXS spectra, we first analyze the spectra of EDTA^4–^ in which scattering through the degenerate resonances of all eight oxygen sites contributes to the scattering intensity. Overall the spectra for resonant excitation show a strong resemblance to π* oxygen K-edge RIXS spectra of acetate^38−40^ and other systems containing σ-bound carboxylate groups^28,41^ underlining the local building block aspect of X-ray spectroscopies. We observe a strong dependence of the spectral shape in dependence on the excitation photon energy. Detuning below the resonance energy largely quenches the emission line at an energy loss of 7.5 eV, whereas the lines at 6 and 10 eV loss remain. Additionally, the emission line at 7.5 eV exhibits enhanced intensity if the incident radiation is polarized vertically (χ = 90°), as does the electronically elastic emission channel at 0 eV. Contrarily, the emission band at 6 eV energy loss shows an inverse dependence on the polarization of the incident radiation, being more intense for horizontally polarized incident radiation (χ = 0°). The polarization dependence lets us assess the orbital character of the emission line at 7.5 eV loss, which is quenched upon excitation energy detuning. Detuned excitation conditions restore symmetry selection rules in systems even with just local symmetry and degenerate core-excited states. The core-excited Jahn–Teller effect induces a symmetry breaking in the core-excited state, which lifts symmetry selection rules for resonant excitation. Such interference effects have been established for RIXS of gas-phase^42,43^ and extended organic systems^44,45^ and have recently been proven useful for investigations of solution phase symmetry breaking.^46^ Symmetry selection rules are modeled intrinsically if RIXS simulations are performed in the so-called delocalized approximation. The simulated RIXS spectrum in this framework is shown in the bottom of Figure 4a, which exhibits no intensity around 7.5 eV energy loss. In contrast, the spectrum in the so-called localized approximation shown at the top in Figure 4a in which interference effects are disregarded exhibits intensity in the corresponding energy loss range with the correct polarization anisotropy. These emission features originate from out-of-plane oxygen lone-pair orbitals with inverse sign at the carboxylate oxygen sites. The decay dipole moment has inverse orientation compared to the elastic channel. As the core-excitation populates an antibonding π* orbital, resulting in parallel moments for the excitation step and antiparallel moments for emission, the scattering amplitude for scattering into the core-holes at the two oxygen sites of each carboxylate group cancel in the symmetric configuration with degenerate core-holes. The core-excited state Jahn–Teller effect induces an antisymmetric distortion of the carboxylate groups, quenching these destructive interference effects and thereby mediating the violation of selection rules in the resonantly excited RIXS spectra. The increased degree of dynamical contributions to the resonant spectra is also reflected in the extent of the vibrational progression of the electronically elastic RIXS channel. Note that the effect is only qualitatively captured by the simulated spectrum in the localized approximation in Figure 4a as the thermal motion of the molecule, solute–solvent interactions, potential ion pairing effects, as well as the dynamics on the core- and valence-state surfaces are not treated explicitly, which would be beyond the scope of this study.

The emission line at 6 eV energy loss can be assigned to in-plane lone-pair orbitals. For these transitions, the emission dipoles add up (at least partially) constructively, avoiding a quenching of the emission line with excitation energy detuning. The same holds for the emission band at approximately 10 eV energy loss, which reflects decay from orbitals with bonding carboxylate π- and σ-character.

The spectral changes induced by the FeEDTA^–^ complex formation are analyzed based on the series of excitation energy dependent experimental and theoretical spectra in Figure 4b,c, respectively. In contrast to the spectra of EDTA^4–^, the emission line related to the lone-pair orbitals disperses strongly with the excitation energy and shifts below 5 eV energy loss. Its width is also increased. To understand these effects, we consider the X-ray absorption profile in Figure 2c. In the case of FeEDTA^–^, we do not detune the excitation energy only from a single resonance, but excite into the ligand field orbitals at lower excitation energies and reach the π* resonances of the peripheral oxygen sites at higher excitation energies. Thus, the final states reached after the RIXS process for lower energies are ligand to metal charge-transfer states characterized by excitations from the occupied orbitals of the ligand into the half filled iron d-orbitals. Their transition energy is of course smaller than for excitations into the higher lying orbitals. Hence, the energy loss of the spectral line increases with the excitation energy. The broadening of the spectral line can be understood considering the role of the carboxylate lone-pair orbitals in the formation of the chemical bond with the iron center. In EDTA^4–^, the carboxylate groups are indistinguishable, whereas in FeEDTA^–^, we can separate the two equatorial carboxylate groups, binding in the same plane as the two nitrogen atoms from the axial ones. This lifts the degeneracy of the lone pair orbitals in the groups, induces a wider distribution of transition energies between the orbitals and thus causes a spectral broadening of the emission line. We illustrate selected orbitals in Figure 4d,e involved in the equatorial and axial bond formation, respectively. Among others, electronic decay from the two upper orbitals to the oxygen 1s orbitals contributes to the spectral intensity of the most intense emission line. Because of the steric hindrance by the ethylene backbone of the EDTA^4–^ ligand, the carboxylate groups are slightly twisted involving both the in- and out-of-plane orbitals in the bond formation. This is nicely seen in the equatorial orbitals in Figure 4d. The topmost orbital reflects the covalent interaction between the out-of-plane oxygen lone-pair orbitals and the iron d_*x*^2^–*y*^2^_ orbital, whereas the second orbital has dominant in-plane lone-pair character with a small d_*z*^2^_ admixture. For the axial carboxylate lone pair orbitals in Figure 4e, the out-of-plane lone pair in the top of the figure does not seem to exhibit sizable metal d-admixture. In contrast, the in-plane lone pair, shown below, strongly mixes with the d_*z*^2^_ orbital. These drastically different interactions between the carboxylate lone-pairs and the metal d-density underline the lifting of orbital energy degeneracy upon complex formation. The two lowest orbitals in Figure 4d,e represent the fully bonding equatorial and axial carboxylate π-systems which contribute only weak spectral intensity at larger energy losses. They seem to be fully decoupled from the iron d-orbitals and are thus not involved in the metal–ligand bond.

We would like to point out, that the separation of the lone-pair emission into symmetry forbidden out-of plane and symmetry allowed in-plane contributions is not as straightforward in the case of FeEDTA^–^. This is induced by multiple aspects. For EDTA^4–^ the selection rules originated from the degeneracy of oxygen 1s core-excited states within the individual carboxylate groups and their violation was coupled to the ability to break the local *C*_2*v*_ symmetry through antisymmetric OCO-stretch modes in the core-excited states. In FeEDTA^–^, the degeneracy of the core-excited states is rearranged to the equatorial and axial oxygen sites individually as well as to the peripheral oxygen sites. Additionally, the symmetry selection rules should be softened already by the twisted structure of the complex. The orbital assignment done in the previous paragraph indicates that the emission from in- and out of plane orbitals with lone-pair character is not as clearly separable as in EDTA^4–^ due to the different covalent interaction with the iron d-orbitals. Additionally, one should consider that the degree of Jahn–Teller induced symmetry breaking also depends on the excited state character. The most comparable situation exists for the strongest resonance of the peripheral oxygen atoms. Here, we detect a shoulder on the high energy loss side of the lone-pair emission line. Under these conditions, the elastic line also exhibits a slight asymmetry indicating a vibrational progression. Compared to EDTA^4–^, both signatures of core-excited state dynamics are quenched by the complex formation. The discussed delocalization of the out-of-plane lone-pairs through the formation of the metal–ligand bond could further suppress the intensity of the emission line. The elastic line is symmetric and the high energy loss shoulder of the lone-pair emission is absent for excitation at the oxygen 1s to ligand field orbitals, indicating an even further reduced extent of core-excited state dynamics possibly also influenced by the steric constraints of the involved oxygen sites which are coordinated with the iron center.

## Conclusion

In conclusion, we investigated covalent interactions between the hexadentate chelating agent EDTA^4–^ and an iron(III) metal center upon complex formation using distinct X-ray absorption and resonant inelastic X-ray scattering signatures at the K-edges of the coordinating ligand sites. Mixing of the nitrogen lone pair orbitals with the d_*x*^2^–*y*^2^_, d_*xy*_, and d_*z*^2^_ orbitals yields the formation of the Fe–N σ-bond, which was probed through new isolated nitrogen 1s X-ray absorption resonances associated with the singly occupied ligand field orbitals in the high-spin FeEDTA^–^ complex. The counterpart, namely the occupied nitrogen 2p-lone-pair orbitals and their delocalization toward the iron center was detected through an intensity reduction and broadening of the lone-pair emission line. Here, the symmetric and antisymmetric combination of the lone-pair orbitals allowed for selective interaction with the d_*x*^2^–*y*^2^_, d_*xy*_, and d_*z*^2^_ metal orbitals. At the K-edge of the carboxylate oxygen atoms, additionally the d_*xz*_ and d_*yz*_ orbitals are accessible through their mixing with the out-of-plane oxygen lone-pairs. We see how the metal–ligand bond formation lifts orbital degeneracy which is reflected in shifts and broadenings of X-ray absorption and emission lines. Additionally, the impact of the steric constraints imposed by the ethylene backbone of EDTA^4–^ mediate coupling of both in- and out-of-plane lone-pair orbitals to the metal d-orbitals. The steric and energetic effects furthermore affect the extent of core-excited state dynamics and interference induced selection rules for specific emission lines.

With our study of free EDTA^4–^ and the sextet FeEDTA^–^ complex, we illustrate how ligand K-edge absorption spectroscopy and RIXS in combination with DFT-based quantum chemical simulations allow one to access the frontier orbitals in high spin systems completely avoiding spin–orbit coupling spectroscopic effects, which drastically complicate the interpretation of L-edge spectra, while carrying a large degree of orbital specific information.

## Supporting Information Available

Iron L-edge X-ray absorption spectrum of FeEDTA^–^ (aq.) and optimized geometries for EDTA^4–^ and FeEDTA^–^ (PDF)
