# Supplementary material for: From the Free Ligand to the Transition Metal Complex: FeEDTA– Formation Seen at Ligand K-Edges
Source: Inorg Chem. 2022 Jun 28;61(27):10321–8. doi: 10.1021/acs.inorgchem.2c00789 (PMC9277664; doi:10.1021/acs.inorgchem.2c00789)
Supplement: Supplementary file 1 — ic2c00789_si_001.pdf [file ic2c00789_si_001.pdf]

Supporting Information: From the free ligand to the transition metal complex: $FeEDTA^-$ formation seen at ligand K-edges

Sebastian Eckert,*,† Eric J. Mascarenhas,‡,† Rolf Mitzner,† Raphael M. Jay,‡,¶ Annette Pietzsch,† Mattis Fondell,† Vinícius Vaz da Cruz,† and Alexander Föhlisch‡,†

†*Helmholtz-Zentrum Berlin für Materialien und Energie GmbH, Institute for Methods and Instrumentation for Synchrotron Radiation Research, 12489 Berlin, Germany*

‡*Universität Potsdam, Institut für Physik und Astronomie, 14476 Potsdam, Germany*

¶*Current address: Department of Physics and Astronomy, Uppsala University, Box 516, SE-751 20 Uppsala, Sweden*

E-mail: sebastian.eckert@helmholtz-berlin.de

L-edge X-ray absorption spectrum of $FeEDTA^-$ In Fig. S1 the X-ray absorption spectrum of aqueous $FeEDTA^-$ is presented for a jet thickness of approx. 2.7 μm and a incidence photon energy bandwidth of approx. 300 meV. A linear solvent absorption background was subtracted.

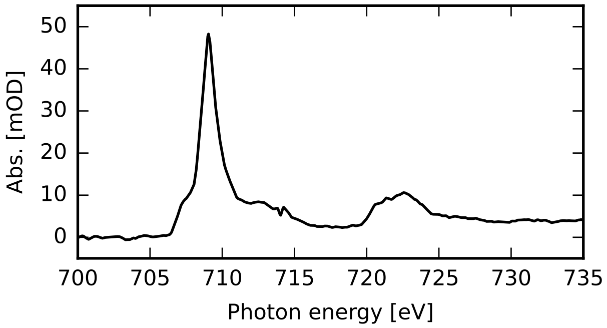

Figure S1: Iron L-edge X-ray absorption spectrum of $FeEDTA^-$.

Optimized geometries of $EDTA^{4-}$ and $FeEDTA^{-}$ In Tab. S1 the optimized geometries of $EDTA^{4-}$ and $FeEDTA^{-}$ (including the coordinated water molecule) in the respective singlet and sextet ground state configurations are given. The structures are illustrated in Fig. S2.

Table S1: Optimized geometries of $EDTA^{4-}$ and $FeEDTA^{-}$

$EDTA^{4-}$				$FeEDTA^{-}$			
Elem.	x	y	z	Elem.	x	y	z
C	-1.4765	1.3245	-0.2686	C	-1.7814	2.9549	-1.0152
C	-0.5845	2.5505	-0.3089	C	-0.6770	3.5063	-0.1540
N	-2.8969	1.6446	-0.2520	N	-2.4262	1.8302	-0.3318
N	0.8271	2.2262	-0.4620	N	0.2782	2.4392	0.1531
C	-3.6626	0.4255	-0.4270	C	-3.1386	0.9579	-1.2589
C	-3.2780	2.3082	0.9832	C	-3.2996	2.2721	0.7607
C	-3.1464	3.8413	1.0495	C	-2.6619	2.2077	2.1392
C	-5.1633	0.5931	-0.7266	C	-3.2990	-0.4254	-0.6557
C	1.3491	1.5536	0.7147	C	1.1097	2.7572	1.3080
C	1.2265	0.0203	0.7842	C	1.6727	1.4802	1.9055
C	1.5740	3.4415	-0.7240	C	1.0978	2.0740	-1.0091
C	3.0229	3.2726	-1.2174	C	0.6691	0.7951	-1.7124
O	3.7156	4.3183	-1.1513	O	-0.1533	0.0339	-1.0818
O	3.3914	2.1685	-1.6731	O	1.1433	0.5406	-2.8116
O	1.2444	-0.6369	-0.2788	O	1.0336	0.4136	1.5976
O	1.1869	-0.4539	1.9483	O	2.6524	1.5295	2.6425
O	-5.8526	-0.4345	-0.5110	O	-2.4931	-0.6962	0.3022
O	-5.5774	1.6791	-1.1869	O	-4.1390	-1.1963	-1.1087
O	-3.2854	4.5063	0.0003	O	-3.1960	2.7984	3.0682
O	-2.9762	4.3076	2.2049	O	-1.6022	1.4862	2.2477
H	1.5985	4.1100	0.1544	H	2.1272	1.9039	-0.6852
H	-3.5701	-0.2502	0.4410	H	-4.1140	1.3636	-1.5422
H	-4.3428	2.1207	1.1531	H	-3.6709	3.2849	0.5843
H	-3.2472	-0.1208	-1.2816	H	-2.5427	0.8423	-2.1665
H	-1.2150	0.7014	0.6053	H	-1.3734	2.5893	-1.9588
H	0.9141	1.9757	1.6353	H	0.4882	3.2250	2.0747
H	-2.7407	1.8787	1.8445	H	-4.1722	1.6164	0.8088
H	-1.2709	0.7240	-1.1583	H	-2.5080	3.7390	-1.2537
H	2.4259	1.7423	0.7632	H	1.9192	3.4486	1.0567
H	1.0566	3.9998	-1.5122	H	1.1271	2.8866	-1.7391
H	-0.8882	3.1666	-1.1587	H	-0.1840	4.3438	-0.6599
H	-0.7415	3.1574	0.6004	H	-1.0783	3.8849	0.7873
				Fe	-0.8489	0.5074	0.7080
				O	-0.6911	-1.3731	1.8787
				H	-1.1505	-2.0763	1.4037
				H	0.2389	-1.6190	1.9541

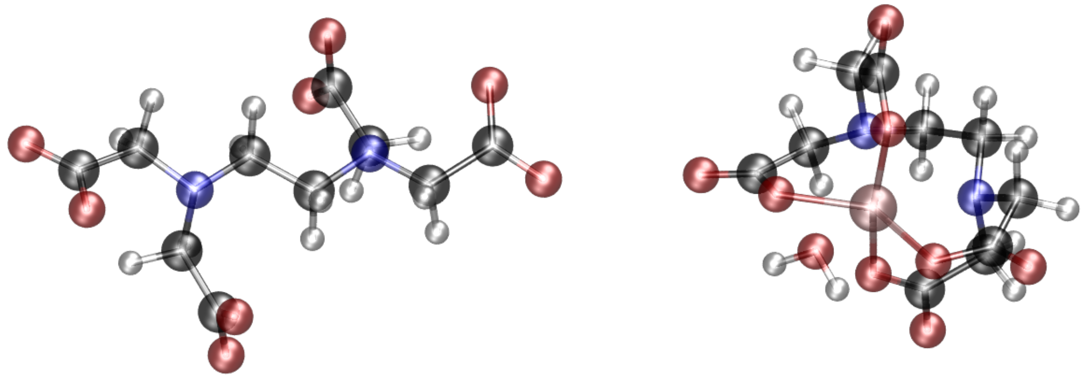

Figure S2: Illustration of the optimized structures of $EDTA^{4-}$(left) and $FeEDTA^{-}$ (right).
